# Evolving practice in global healthcare: Remote physics support for low‐ and middle‐income countries

**DOI:** 10.1002/acm2.13914

**Published:** 2023-01-31

**Authors:** Shada Wadi‐Ramahi, Benjamin Li, Fayez Waqqad, Abdelatif AlSharif

**Affiliations:** ^1^ Medical Physicist, Radiation Oncology Department, Hillman Cancer Center University of Pittsburgh Medical Center Pittsburgh Pennsylvania USA; ^2^ Radiation Oncology Resident PGY4, Department of Radiation Oncology University of California San Francisco San Francisco California USA; ^3^ Medical Physicist, Radiation Oncology Center King Abdullah University Hospital Irbid Jordan; ^4^ Medical Oncologist and Director, Radiation Oncology Department Afia Nuclear Medicine and Radiation Oncology Amman Jordan

**Keywords:** Global Healthcare, LMICs, physics support

## Abstract

The COVID‐19 pandemic has disrupted traditional onsite support for radiotherapy clinics in low‐ and middle‐income countries (LMIC). Clinics there have struggled to commission new techniques and receive onsite training for their staff. We sought to evaluate whether an offsite approach could fill this gap at a clinic in Jordan by requesting a clinical audit and attempting to commission volumetric modulated arc therapy (VMAT). Over 13 months, a consultant provided remote support for a radiotherapy center that had already obtained treatment equipment and licenses. The consultant began by conducting a virtual audit, using a remote login to the center's R&V and TPS, to identify any gaps in the clinical workflow. Suggestions for improving the clinical workflow were proposed, and change implementation was tracked through emails, social media apps, and video conferencing. An extensive table outlined the commissioning process, including all measurements to be done. Social media apps and shared documents were used to track measurements and analysis. The lack of person‐to‐person interaction in this new remote‐support ecosystem created conflicts; we have highlighted some of these, as well as their resolution and the lessons learned from them. The virtual audit identified gaps categorized as machine QA, treatment plan review, and treatment delivery processes. Following the implementation of the proposals, motion management was added, and machine QA became more comprehensive. VMAT was commissioned using the reports of the AAPM and the IAEA. The main challenges for remote support were time difference, establishing an appropriate form and frequency of communication, tone of voice used in messages, and buy‐in from local staff. This evolving practice will enable medical physicists to use modern, multimodal remote communication pathways to effectively transfer knowledge to centers in LMICs. The audit–proposal–improvement pathway for remote support can be incorporated to help others while avoiding the pitfalls we faced.

## 1 INTRODUCTION

Advancements in low‐ and middle‐income countries (LMICs) have been implemented largely through the help of professionals from developed countries and usually at a significant cost shouldered by one or both sides. Cost is mostly associated with sending and accommodating a group of experts to provide training at the receiving institution or sending a team of professionals from the LMIC institution to a host institution.^1^, ^2^, ^3^, ^4^ In this way, many collaborative partnership agreements have been signed to facilitate the flow of expertise between institutions in advanced countries and their counterparts in LMICs, as captured by Khader et al.^5^ In the white paper published by Abdel‐Wahab et al.,^6^ the authors recognized that in LMICs where the number of machines is adequate, there still lies the challenge of implementing the technology and training professionals. They identified the use of modern telecommunications, including virtual collaboration, as an asset that can bridge the technology transfer gap.

While the value of physical interaction and on‐site training is not in question, the COVID‐19 pandemic, which shut down many borders and almost halted international travel, forced us to think creatively about how to provide training and support for the LMIC institutions that request it. Remote access through a secured network to actively engage professionals with reviewing plans and treatment and auditing activities has been discussed before.^7^, ^8^ One group^9^ provided good‐practice guidelines for remote medical consultation, while another discussed the adaptation of education and research on telehealth^10^ as lessons learned in the era of COVID‐19. A recent paper^11^ discussed the successful implementation of a distant‐learning clinical research mentorship between Canada and Ghana.

Jordan is a country situated at the northern part of the Arabian Peninsula. Its 2020 census put the population at a little less than 11 million (median age 23.5 years).^12^ In 2020, there were close to 12,000 new cases of cancer in the country, and about half that number passed away from the disease.^13^ The most prevalent cancer in females is breast followed by colorectal, whereas in men it is lung followed by colorectal. There are four operational radiation oncology departments in the country, with only two offering intensity‐modulated radiation therapy (IMRT) treatment and one offering volumetric modulated arc therapy (VMAT) treatment.^14^

This work describes the effort to provide a full clinical audit and support for commissioning VMAT in one of Jordan's cancer centers in a remote‐support, multi‐modal ecosystem. The hardships and lessons learned are highlighted, as this experience is new in our professional field. We present the work as a proposed roadmap that can be adapted for future remote consultation work.

## 2 METHODS

Initial contact from the cancer center took place in spring 2019 by the CEO of the hospital. The details and scope of work were discussed over few months and two short visits (3–5 days) that occurred in summer and fall 2019 to assess the situation. During these initial visits, the expert met with the CEO, the director of the cancer center, and the staff. The future direction of the center, expectations and readiness for audit, and improvements were discussed. As a first step, it was crucial for the consultant to observe the clinical workflow and learn how things were done. The initial plan was to conduct multiple short visits over 2020 to implement improvements to current practices before commissioning VMAT. The plan also included being present during the center's first patient VMAT planning and treatment.

The plan changed dramatically in early 2020, with the COVID‐19 pandemic. More time and effort had to be put into a remote support system, including clarifying the mode, frequency, and level of communication, re‐organizing the work with clear instructions concerning the local staff's roles and responsibilities, as well as those of the consultant, and agreeing on methods for follow‐up and work approvals.

Various social media apps were used for instant communication with video conferencing and communication through voice messages, texts, and shared documents. The choices made were dependent on what was available in Jordan. Social media apps that allowed for groups to be formed were used for offline communication with interested parties working on a certain project. The apps had to allow the sharing and saving of documents and attachment of video or audio messages. Finally, it was agreed that communication by email would be the official mode, and through it, we communicated the goals and actions that were agreed on to the center's administration.

### 2.1 Virtual audit

In the two short visits in 2019, the consultant had the chance to shadow the center's two physicists and observe some of the linear accelerator (linac) QA work. However, the majority of the audit took place remotely. The consultant requested access, through a secured VPN connection, to the center's records and verification system as well as to the treatment planning system. The audit took a form similar to an extensive weekly chart check and included an offline imaging review. This allowed for the assessment of image‐guided radiation therapy practices. The review was conducted over the span of 8 weeks and included all patients treated on the two linacs. Most cases were palliative, and among the curative cases, breast was the most common cancer, followed by gastrointestinal and genitourinary. For curative cases, the center used three‐dimensional conformal radiation therapy (3DCRT) treatment, and some of this (∼15%) had been done using IMRT at the time of the audit. After each weekly audit was concluded, an email with the findings and points for improvement were communicated to the chief therapy technician and medical physicists, and this was followed up by a phone call.

The clinical management of patients was investigated across each step of the workflow from the CT simulation setup to contouring, treatment planning, and treatment delivery. This was done by going over CT simulation immobilization and setup notes for each clinical site and reviewing the CT scans to verify positioning. The 3DCRT plans were reviewed, and the center's team was asked to select IMRT cases representing head and neck and prostate to be reviewed by an external radiation oncologist. A total of eight cases were selected. A regionally renowned radiation oncologist was enlisted by the consultant as a volunteer to review the contours and the plans’ objectives for each IMRT case. The review was done asynchronously and concluded with a document describing the feedback of the reviewer by email.

**FIGURE 1:**
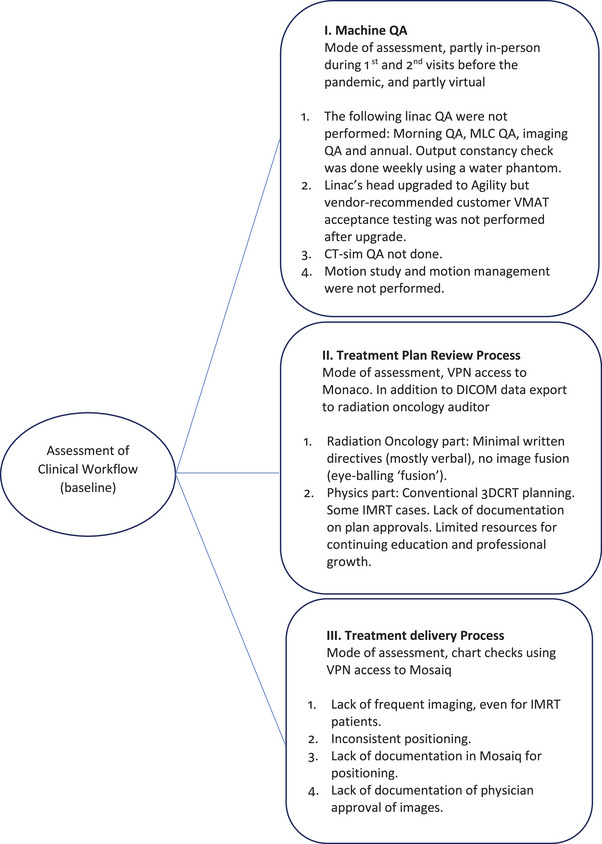
The three major arms of the review audit to assess the site's workflow.

The virtual audit continued by looking at the center's documented QA practices, and discussions ensued with the physics team using social media app messaging. The machine QA audit established a baseline for the consultant, which helped identify items for improvement and changes in practice. One such practice that underwent drastic change was the measurement of linac output, which at that time was done on a weekly basis using a water tank. Items for practice improvement were put into three categories, namely machine QA, planning review, and treatment delivery. Figure 1 details the items under these categories.

### 2.2 VMAT commissioning

The importance of linac output verification by a third party was highlighted, and the consultant requested having the linac enrolled in the IAEA TLD audit.^15^ A review of the available 3D motorized water phantom, chambers, and other tools for measurement and QA was carried out with the help of the local physics team. Recommendations were made for purchasing certain detectors and a thorax phantom, which were essential for carrying out the tasks at hand, and these recommendations were accepted by the administration. The consultant also requested access to the initial acceptance testing, and commissioning documents were sought for review. It was agreed with the local physics team that the linac would go through its first‐ever annual QA test. A detailed table was compiled with the measurements to be used for the commissioning process and the verification with the thorax phantom. Next to each task was the role of the local physics staff members and that of the consultant as well as the frequency and means of communication.

## 3 RESULTS

The virtual audit and implementation of the proposed changes took 13 months. One hurdle was the inability of the linac vendor to send a team to perform acceptance testing of the newly installed linac head as a result of travel restrictions; there was also the waiting time required for the IAEA TLD audit for the linac output.

**FIGURE 2:**
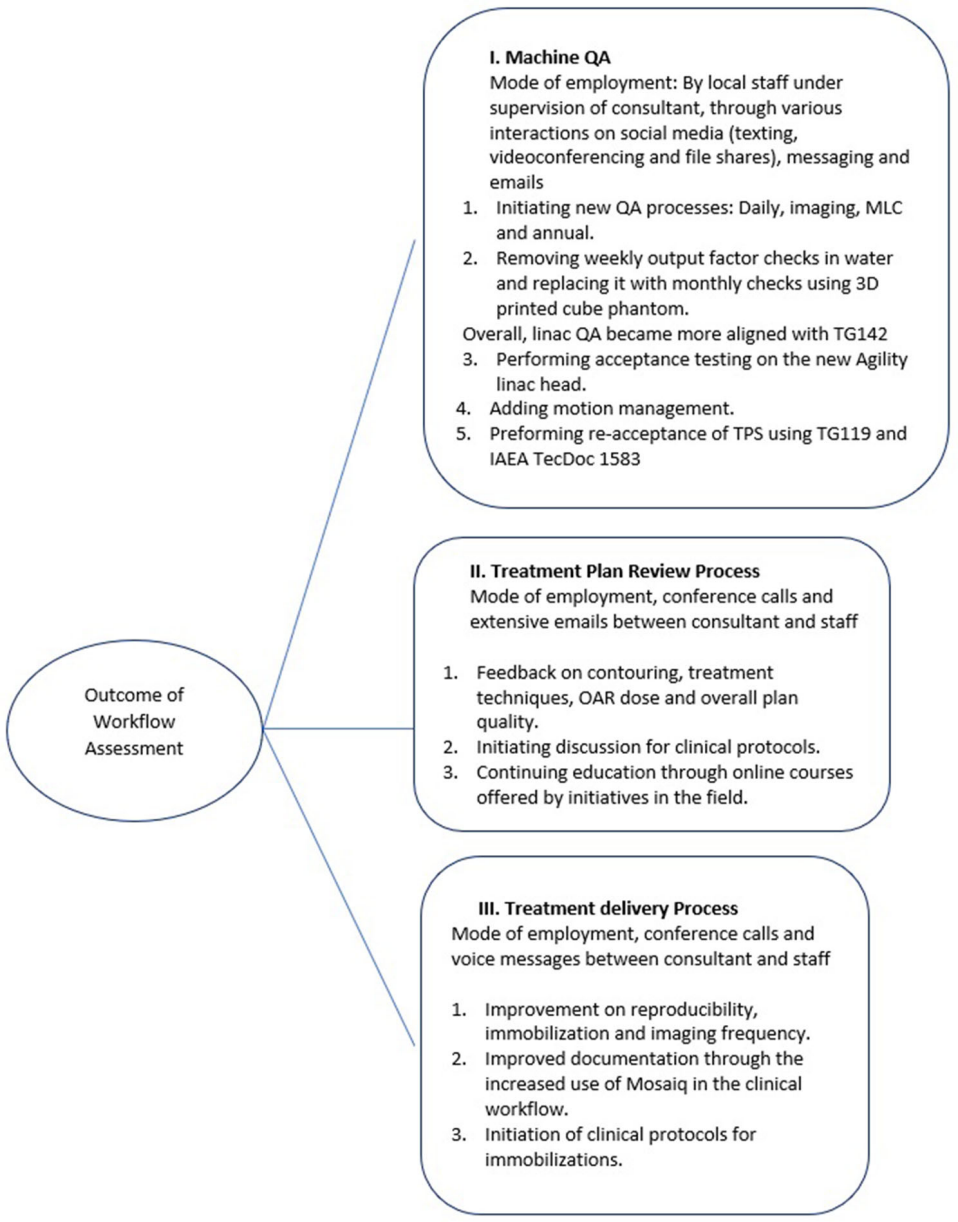
Implemented changes in the three arms of the review audit.

Implementing the recommended changes in the three categories of machine performance QA, treatment plan review process, and treatment delivery process resulted in an improved clinical workflow. Figure 2 summarizes these improvements.

### 3.1 Machine performance and QA

**FIGURE 3:**
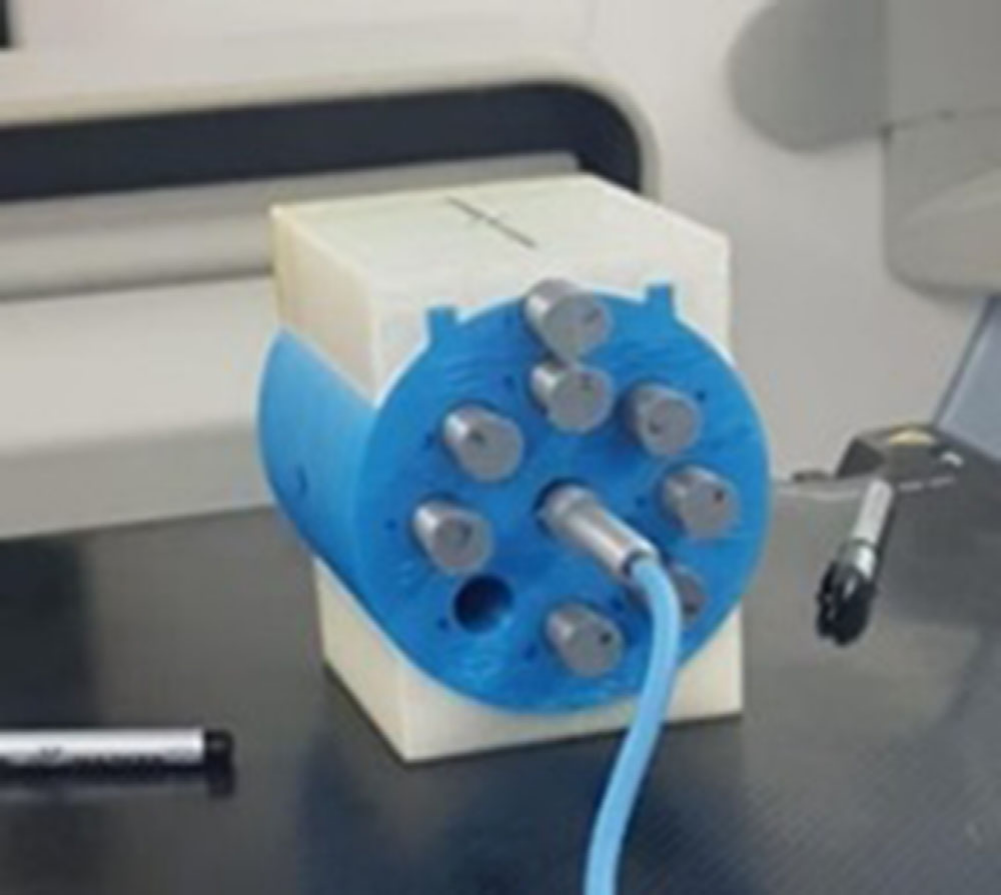
3D printed phantom for the monthly output measurements.

Major improvements in the linac QA process were made by adding the multileaf collimator and imaging QAs. In addition, the weekly output constancy measurements, which were being done in a water tank, were stopped and replaced by a monthly output measurement carried out with a 3D‐printed phantom. The 3D‐printed phantom was initially CT‐scanned to look for heterogeneities in printing and verifying the fit of the ion chamber slots. As shown in Figure 3, it was composed of multiple slots for inserting a 0.6 cc chamber at various depths. For each slot not used, a rod of identical dimensions and material was inserted to plug the hole. The baseline data for this phantom were taken after performing reference dosimetry based on TRS‐398^16^ during the annual QA. After all improvements had been completed, the linac QA process was more aligned with TG142.^17^

### 3.2 CT simulation and treatment planning process improvement

Another major implementation was adding four‐dimensional computed tomography (4DCT) clinical workflow for lung patients. An illustration of the 4DCT concept using a locally bought moving doll was provided to the clinical team, followed by a thorough discussion on the technique. One of the staff (chief RTT) consented to be a volunteer for a 4DCT scan that would later be used for further testing of the TPS. The team then developed a clinical protocol for patient selection, justification, and the 4DCT simulation workflow. Imaging using 10‐phase binning was adopted. The concept of internal target volume (ITV) to encapsulate the target motion was used for motion management. The patient population seen by the clinic did not justify capital investment in more involved motion management techniques.

### 3.3 Treatment delivery

Clinical protocols discussing the frequency and mode of imaging on the linacs for palliative and curative cases were discussed with the staff, and protocols were encouraged to be written. Feedback to the staff, including the chief therapist, was initially given after each weekly chart review. The review pointed out heterogeneity in setup and imagining frequency and demonstrated the need for standardized practice. The implementation of the newly established clinical protocols with regard to imaging frequency and types of imaging, as well as the presence of the treating physician for certain cases, was confirmed in subsequent reviews. These chart reviews were done remotely using secured access to the center's record and verification system. Discussions with the chief therapist were carried out using a social media app that allowed free, over‐the‐internet calls.

### 3.4 VMAT commissioning

The IAEA TLD audit service showed that the linac output was within acceptable limits, and the center's administration was advised to adhere to the frequency of the IAEA TLD auditing. Required measurements were carried out by the local physics team per the agreed upon table. Discussion of results with the consultant took place frequently using social media apps that also allowed video clips to be uploaded for review, if needed. Formal documentation of the data was done by email. The performance of the treatment planning system^18^ was verified through the implementation of both TECDOC1583^19^ and TG119.^20^ The IMRT thorax phantom^21^ was used during the TPS verification as well as for treatment planning training and end‐to‐end (E2E) testing. Twenty different plans were chosen for the E2E testing covering various disease sites, such as brain, head and neck, lung, and male and female pelvis. The gamma analysis for the 20 plans showed that 2%/2 mm analysis was passing with a rate >90% for 15/20 plans. However, for the center's clinical practice, the staff opted to choose 3%/3 mm gamma analysis and a passing rate of >90%.

### 3.5 Where do we go from here? Continuing education and training

Realizing that continuing education is crucial to building expertise and improvement, the consultant connected the center to a global initiative that would provide virtual educational courses for all the staff in the radiation oncology department. This initiative has resulted in several focused training courses being conducted with proven results.^22^, ^23^ Each course aims to cover a certain topic and runs over a 12‐week period. The center, at the time of writing, has completed its participation in the SRS/SBRT course that was offered by this imitative, which started in November of 2021. By connecting the staff to a venue that provides continuing education, the loop, as mentioned in the white paper,^6^ has been closed.

## 4 DISCUSSION

Implementation of the requested clinical audit and VMAT technique was successful. Cancer patients in Jordan now have access to another cancer center that provide motion management and VMAT. As a side result, the local physics team has gone through an extensive commissioning process that included performing a TLD audit on their linacs and established their annual QA process.

### 4.1 Hurdles, conflict management, and lessons learned

The measurement‐heavy and sensitive work was marred by a variety of hurdles and miscommunication that at certain points halted the work altogether. Table 1 discusses the hurdles faced during this project, which were partly caused by COVID‐19 restrictions. All points of conflict were based on miscommunication, and one was due to inappropriate professional behavior.

**TABLE 1:** Steps for remote commissioning

Steps to be done sequentially	Responsibilities and mode of communication	Hurdles and conflict situations	Parallel work/lessons learned
1. Performing acceptance testing on the Versa head. – Side project was taking measurement for the linear accelerator's first ever annual QA.	Local staff with local agent of the linac vendor. Heavy on email communication with the consultant for documentation purposes and few calls to verify acceptance testing is going per vendor's documentation.	The communication with the vendor started early on, as soon as the consultant found that the Versa head was installed but acceptance was not performed. However, movement of vendor's engineer was restricted due to COVID19, **hurdle#1**. Took months from scheduling to actual acceptance testing. Hospital administration was highly involved to solve bureaucratic and financial issues stemming from the fact that acceptance testing is being requested 2 years after new head was installed, **hurdle#2**.	The interim time waiting for the acceptance testing was used productively to: Continue the improvement in the clinical workflow and process. Perform annual QA. Perform measurements for IAEA TLD audit for the linac output.
2. (re)Performing acceptance testing of the TPS using TRS1513 and TG119.	Local physicists with heavy involvement (remotely) of the consultant with frequent video calls using: Messenger and Google meet. As well as heavy use of voice and written messages using WhatsApp. Emails were used infrequently, and reserved to communicate the progress to the administration at certain checkpoint.	**Hurdle#3**: The local site did not have a heterogenous phantom to be used for the project. The hospital's administration, upon recommendation from the consultant, bought a thorax phantom from CIRS to be used for acceptance testing and commissioning work. **A heavy point of conflict**. Local physicists at first, understandably, did not want to do the task, arguing that the TPS has already done through acceptance testing years ago and their work was audited by a 3rd party consultant in recent years. In addition, they follow IAEA recommendations and not TG119. Work almost halted for a while pending resolution. Eventually the consultant argued that because the hospital did not fully perform acceptance testing per TRS1513 (due to lack of a proper phantom) that repeating the work is warranted	Point of conflict had to be solved satisfactorily, which came at with many**lessons learned**: 1 Communication style: Local physicists felt ‘talked down to’. Consultant owned up to miscommunication and later changes in the consultant's team occurred. 2 Communication content: Local physicists hold graduate degrees and one of them is certified by the IMPCB. However, they were excluded from certain discussions, especially the one for rationale of repeating certain tasks. Expert acknowledged this miscommunication from their part. 3 Communication style: Consultant complained about passive aggressive (written) Behavior
3. Commissioning and E2E QA	Local physicists with heavy involvement (remotely) of the consultant with frequent messaging on WhatsApp and use of Messenger video calls when needed.	**Hurdle#4**: Agreeing on results for the Thorax IMRT phantom. Agreeing on measurements to be done. **Point of conflict**: Tone of communication as perceived by local staff – authoritative and not cooperative	**Lesson learned**: Include local physicists with the thought process and reach agreements on tasks.
4. Training on VMAT	Linac vendor provided remote training on VMAT planning to the local staff. No involvement of the consultant.	**Hurdle#5**: Due to COVID‐related travel restrictions the application specialist from the vendor was not able to travel to provide training on‐site. A remote training was then agreed on.	
5. Establishing gamma analysis protocol	Local physicists with heavy involvement of the consultant with frequent emails. QA measurements of 20 plans representing different anatomical sites were sent by email and discussed by emails.	N/A	Refreshing to have worked out all issues of communications and have the project end on very positive note from all sides.
6. Go live	January 2021: First VMAT treatment.		

#### 4.1.1 Communication with the local physics team

- Exclusion of the local team from decision making
- Communication pathways

Initially, the local physics team felt excluded from the decision‐making process. As a result, there were many challenges to the proposed changes. The second issue concerned the communication pathways. The consultant had always complied with including the center's administration in all email communications, even including clinically oriented measurement‐only emails. The conflict came to the fore when the physics team voiced its concerns about the need to loop in the administration for everything and asked for trust and autonomy for decisions related to pure physics work, such as machine QA.

The consultant also agreed with the physics team about its members’ involvement in decision making. It was agreed that any proposals for change or improvement would be discussed with the physics team over social media and then presented to the administration by the consultant as a representative of the physics team.

#### 4.1.2 Coordination of communication and point of contact

Due to the large time difference (+7 h) and differences in the weekend schedule, there was a need to have someone geographically closer to answer questions and provide feedback and directions while the local physicists were in the clinic measuring data. The consultant requested the help of a physicist located in the region. It was later realized that the enlisted regional physicist had been requesting additional tasks or asking for repeats without coordinating with the consultant, which conflicted with the previously agreed upon timeline and task schedule. This led to a conflict between the enlisted regional physicist and one of the local physics staff. This conflict escalated to improper professional behavior. Once this was realized, conflict resolution began by recognizing the need to own up for the miscommunication and the conflicting information on the consultant's side, and both sides acknowledged their actions. Both sides (the consultant and the center's administration) made changes as a result of this issue that included changes to the personnel.

Keeping an open line of communication and maintaining transparency with administration and staff are challenges in regular (in‐person) work environments, and these were compounded by virtual discussions taking place through social media, voice messages, and emails. The written word is known to lack emotion, and can hence be easily misunderstood. What was new to us, although it is intuitive in hindsight, was that the intent of voice messages could also be misunderstood depending on the inflection of the voice. As one records a voice message containing technical details, the voice will most likely be animated or devoid of emotions, and it can thus come off as condescending, careless, or uninvolved. Video or call conferencing had the least miscommunication associated with them, yet they were not always practical due to timing and weekend differences, and they were used the least.

## 5 CONCLUSION

We demonstrated an audit–proposal–improvement process for successful remote commissioning. To the best of the authors’ knowledge, this was the first attempt in Jordan, and the region, in which remote physics consultation was provided to audit clinical practice and to help in commissioning new techniques.

While the in‐person commissioning process can usually be accomplished in a few weeks, the remote process took 13 months due to multiple hurdles. In the evolving practice of global knowledge exchange, pioneers such as Radiation Knowledge, RCC, and the Global Health Catalyst^24^, ^25^, ^26^ provide the proper infrastructure and environment for true global education and training. We believe that the audit–proposal–improvement pathway for remote support and knowledge transfer can be incorporated with reduced cost to help other radiotherapy clinics in LMICs, and it can possibly be generalized to other technical specialties to help other clinics around the world. To make the best use of time and expertise on both sides, care should be taken to ensure clear communication and to maintain professionalism.

## AUTHOR CONTRIBUTION

Shada Wadi‐Ramahi, Fayez Waqqad, and Abdelatif AlSharif contributed to the design and drafting of the work. Shada Wadi‐Ramahi and Fayez Waqqad contributed to the acquisition and analysis. Benjamin Li contributed to the interpretation of the data in the remote (global) context and provided very critical and detailed review of the draft and instituted fundamental changes to the manuscript and its subsequent revisions. All authors are aware of each other's contribution and all authors take full responsibility for the presented data.

## CONFLICT OF INTEREST

The authors declare no conflict of interest.

## Data Availability

Data sharing not applicable to this article as no datasets were generated or analyzed during the current study.
